# Rett Syndrome. A Review with Emphasis on Clinical Characteristics and Intervention

**DOI:** 10.1100/tsw.2006.249

**Published:** 2006-12-06

**Authors:** Meir Lotan, Bruria Ben-Zeev

**Affiliations:** Israeli Rett Syndrome Association National Evaluation Team and National Rett Syndrome Clinic Chaim Sheba Medical Center, Ramat-Gan, Israel

**Keywords:** Rett syndrome, clinical characteristics, review

## Abstract

Rett syndrome (RS) is a genetic disorder affecting mainly females. In the majority of cases, it is caused by a mutation in MECP2, an X-linked gene, and considered the most common multidisabling genetic disorder in females after Down syndrome. This article is an introduction to RS. It presents the basic understanding of common characteristics typical of this disorder, and the variants from the classical expression of RS. The present article will review the current literature on RS, specially focusing on the clinical characteristics of the disorder. The intention of the article is to set a clear, up-to-date picture of the individual with RS to prepare the clinician for their future meetings with this population.

## INTRODUCTION

Rett syndrome (RS) was first described as a clinical entity in the German literature in 1966[1]. During the period 1966–1969, Andreas Rett (1924–1997) reported on 22 other cases of females with this disorder and kept on lecturing on this topic throughout Austria and Germany[2]. In 1982, he published a second article on the efficiency of music in the management of individuals with this disorder, again in the German language[3]. The awareness to this disorder in the English medical literature was established by Bengt Hagberg and his associates from Sweden in 1983 with a description of 35 individuals with strikingly similar clinical features of: “progressive autism, loss of purposeful hand movements, ataxia, and acquired microcephaly”[4]. Since then, it was found that RS affected mainly females and it was found in a variety of racial and ethnic groups worldwide with a versatile clinical phenotype[5]. The estimated incidence of RS is 10/100,000 females[6], although higher incidence rates have been reported[7,8,9]. It is considered to be the second most common cause, after Down’s syndrome, of multidisabling genetic disorders in females[10,11].

RS is a severe neurodevelopmental disorder characterized by the loss of intellectual functioning; fine and gross motor skills, and communicative abilities; deceleration of head growth; and the development of stereotypic hand movements, occurring after a period of apparently normal development. Individuals with RS often develop seizures, a disturbed breathing pattern with hyperventilation, and periodic apnea, scoliosis, growth retardation, and gait apraxia[12]. The next section will describe the main diagnostic feature for this population.

## DIAGNOSIS

RS results from an X-linked dominant mutation in MECP2 gene[13,14]. To date, mutations in this gene can be found in about 90% of females with the classical phenotype of this disorder[15]. Despite the genetic high specificity and sensitivity, RS is still considered a clinical diagnosis characterized by a specific developmental profile that should meet certain clinical diagnostic criteria[16,17]. The diagnostic criteria for classical and variant RS as derived from the paper by Hagberg et al.[17]:

**Inclusion criteria**

- Apparently normal pre- and perinatal history
- Psychomotor development normal during the first 6 months (may be delayed from birth)
- Normal head circumference at birth
- Postnatal deceleration of head growth (most individuals)
- Loss of purposeful hand skills between 0.5–2.5 years
- Stereotypic hand movements
- Evolving social withdrawal, communication dysfunction, loss of acquired speech, cognitive impairment
- Impaired or deteriorating locomotion

The first three clinical criteria may not be applicable to severely affected females; other criteria will not apply to those who are mildly affected and are only identified due to positive findings of mutations in MECP2.

**Supportive criteria**

- Breathing disturbances while awake
- Bruxism
- Impaired sleeping pattern from early infancy
- Abnormal muscle tone accompanied by muscle wasting and dystonia
- Peripheral vasomotor disturbances
- Progressive scoliosis, or kyphosis
- Growth retardation
- Hypotrophic small and cold feet and/or hands

**Exclusion criteria**

- Organomegaly or other evidence of a storage disorder
- Retinopathy, cataract, or optic atrophy
- History of perinatal or postnatal brain damage
- Identifiable inborn error of metabolism or neurodegenerative disorder
- Acquired neurological disorder due to severe infection or head trauma

### Genetic Diagnosis

In 1999, mutations in the MECP2 gene were found in patients with RS and these aberrations were considered the main cause for this disorder[13,14]. RS is inherited in an X-linked dominant manner. Approximately 99.5% of cases are sporadic, resulting from a *de novo* mutation in the embryo with RS; 0.5% of the cases are single occurrences in a family, inherited either by a mutation carrier mother or by gametic mosaicism. In most familial cases, the carrier mother has favorably skewed X-chromosome inactivation that results in her being unaffected or only slightly affected[18]. In the case of a carrier mother, the recurrence risk is 50%. If a mutation is not identified in leukocytes of the mother[19], the risk to sibs of the proband is below 0.5% (since germline mosaicism in either parent cannot be excluded). Of note, because of this fact, it is appropriate to offer prenatal diagnosis to couples who have had a child with RS or mental retardation due to a MECP2 mutation whether or not the disease-causing mutation has been identified in the mother (since it is not possible for the father to carry pathogenic mutation and be normal)[20].

## Genotype-Phenotype Correlations

The below-mentioned articles are investigations that attempt to disclose a genotype-phenotype correlation. These studies have so far yielded inconsistent results. It seems that a far better understanding of MECP2 function is required, and further controlled studies are needed before valid conclusions can be drawn about the effect of mutation type on the phenotypic expression.

- Leonard and associates[21] began such an analysis by evaluating phenotypes of different individuals with the same mutation (R133C). These patients had a less severe phenotype consistent with *in vitro* functional studies, demonstrating that R133C does not impair binding to DNA.
- Amir et al.[14] found a positive correlation between truncating mutations and breathing abnormalities, whereas scoliosis was more common in patients with missense mutations. Neither the overall severity score nor other parameters (age of onset, mortality, seizures, and somatic growth failure) correlated with the type of mutation.
- Cheadle et al.[22] found significantly milder disease in patients with missense mutations than in those with truncating mutations. They also found that late truncating mutations produced milder phenotypes than early truncating mutations ($p = 0.0190$).
- Charman et al.[23], based on a population of 116 individuals diagnosed with classic RS and 19 patients with an atypical presentation, claim that individuals with late truncating mutations had a less-typical presentation than cases with missense and early truncating mutations, presumably reflecting greater residual function of MECP2 protein. Individuals with early truncating mutations had a more severe outcome than cases with missense and late truncating mutations.
- Cheadle et al.[22] and Huppke et al.[24] both reported several patients with the same mutation, but different phenotype; findings suggesting that factors other than mutation type influence disease severity. One such factor is the pattern of X-chromosome inactivation (XCI); females who have a mutation, but have favorably skewed XCI, may have milder symptoms or none at all[14,25].
- Several of the MECP2 mutations found in males with mental retardation are typically missense or late truncating mutations that have not been identified in females with RS. Missense mutations such as A140V result in partial inactivation of the protein. Therefore, it causes mental retardation in males but very mild cognitive impairment in females[26].

## Biochemical Diagnosis

Laboratory studies have not been found useful in establishing the diagnosis through biochemical indicators since no consistent abnormalities have been found. Although alterations in CSF biogenic amine metabolites and beta endorphins have been reported in RS, none of the finding was consistently abnormal[27].

## STAGES OF RETT SYNDROME

Individuals with RS show a pre- and perinatal period with apparently normal developmental progress for the first 5–6 months of life. The birth head circumference is within the norm, with subsequent deceleration of head growth, usually leading to microcephaly emerging at around 4 months postnatally[28,29]. At this stage, minor developmental abnormalities are evident in the majority of these children, such as mild hypotonia, poor suck, a weak cry, and a calm disposition. Such minor signs are intricate to detect even by experts in developmental disabilities[30,31]. The development of RS is usually described in four stages[12,32]:

- **Stage I: Early Onset Stage —** Between 3 months and 3 years, there is reduction or loss of acquired skills such as purposeful hand function, vocalization, and communication skills. The girls then enter a short period of developmental stagnation.
- **Stage II: Rapid Destructive Stage —** The second stage is detected when the child is around the ages of 1–4 years. This stage is usually short (duration: weeks to several months). It is characterized by rapid regression in language and motor skills. One of the characteristics of this stage is extreme screaming and crying episodes by 18–24 months of age[33]. Additional characteristics include autistic-like features, panic-like attacks, bruxism, episodic apnea and/or hyperpnea, seizures, gait ataxia and apraxia, tremors, and acquired microcephaly. The emergence of hand stereotypical mannerisms is the prominent feature of this stage. Intermittent esotropia is common and vasomotor changes are often noted, especially in the lower limbs, as well as deterioration in head growth. After this period of rapid deterioration, RS becomes relatively stable.
- **Stage III: Plateau Stage —** The third stage is usually apparent at preschool age to adulthood. Apraxia, motor problems are more prominent during this long stage. The individual with RS will show less irritability and enhanced awareness of her surrounding. This stage usually can last for decades and enables the person to gain achievements in various fields.
- **Stage IV: Late Motor Deterioration Stage —** This stage is characterized by reduced mobility (those who were never ambulant move from Stage II to IV). Despite the motor deterioration at this stage, there is no decline in cognition communication or hand skills. At this stage, individuals with RS might develop dystonia, and foot and hand deformities as they grow older.

The principal characteristics of RS and their developmental pattern indicate that abnormal development of the cortex during infancy and dysfunction of subcortical regulator systems, brain stem, basal forebrain nuclei, and basal ganglia is apparent. Due to functional disturbances in RS, such as breathing, cardiac rate, swallowing, peripheral vasomotor disturbances, sleep, bowel motility, and salivation, brain stem involvement is considered significant[34,35]. The cardiac and breathing abnormalities can be based on dysregulation of autonomic tone with failure to regulate vagal (parasympathetic) tone and respiratory rhythm[36].

The hallmark of RS is the intense, and almost continuous, hand stereotypic movements[37], which develop after a period of purposeful hand use at infancy. Those hand mannerisms include hand wringing, hand washing, clapping, patting, or other hand automatisms, during waking hours[12]. A jerky truncal and an ataxic gait pattern is another prominent feature. Other features include breathing dysfunction, EEG abnormalities, spasticity, peripheral vasomotor disturbance, scoliosis, and growth retardation. RS may overlap with other disorders[38,39] in which case the appropriate diagnosis might be delayed.

## VARIANT PHENOTYPES OF RETT SYNDROME

Many variants have been described that may display different clinical pictures than the one presented by the individual with classical RS[40]. Individuals with RS might present a broad clinical variability that is termed “atypical forms”. These variations may be either milder or more severe than the classic RS phenotype[10,41].

**Inclusion criteria**
- At least 3 of the 6 main criteria mentioned above
- At least 5 of the 11 supportive criteria mentioned above
- Absence or reduction of hand skills
- Reduction or loss of speech (including babble)
- Hand stereotypes
- Reduction or loss of communication skills
- Deceleration of head growth from early childhood
- Regression of human interaction followed by recovery

**Supportive criteria**
- Breathing irregularities
- Air swallowing or abdominal bloating
- Bruxism
- Abnormal locomotion
- Scoliosis or kyphosis
- Lower limb amyotrophy
- Cold, discolored feet, usually hypotrophic
- Sleep disturbances, including night-time screaming
- Inexplicable episodes of laughing or screaming
- Apparently diminished pain sensitivity
- Intense eye contact and/or eye pointing

Variants of RS might appear with severe or a milder form. The severe variant has no normal developmental period; individuals with a milder phenotype experience less-dramatic regression and milder expression of the characteristics of RS[42]. In this group, one might find the “Forme fruste” (FF)[43] or the preserved speech variant (PSV). Although initially thought to be a disorder exclusively affecting females, males with a Rett-like phenotype have been reported occasionally[44,45]. As MECP2 mutations and their particular effects are better understood with time and research, characterization of the various phenotypes associated with mutations of this gene should be possible. The varieties of RS are further discussed below.

- **Early Onset RS/Congenital RS** — This is a more severe variant. No period of grossly normal development occurs, and early manifestations include congenital hypotonia and infantile spasms “early epileptic form”[19,46]. The child showing infantile spasm may later develop a severe manifestation of RS or a classical one[12,47]. In a second variant, the early “normal development” is completely absent, making the diagnosis of the child as an RS client difficult. This type is referred to as “congenital RS”[11]. So far, no MECP2 mutations have been diagnosed in the early epileptic forms and CDKL5 mutations (an X-linked gene) were found in some[19].
- **Forme Fruste (FF)** — A known variant of RS with milder clinical features. In this form of RS, the clinical signs appear at an older age (around age 2–3 years or up), after the child has already developed expressive language. It is not always easy to connect this regression to the classical appearance of RS, but more and more manifestations of RS are usually apparent with the passing years[10]. Individuals with FF RS usually present milder reduction in head circumference, superior functional hand use, and sometimes the hand mannerisms show a subtler appearance[43].
- **Preserved Speech Variant (PSV)** — These children experience a gradual regression that begins after the third year. They do lose part of their purposeful hand use and can develop seizures, although they retain some speech and the ability to walk[48]. In this form of RS, partial language is preserved; it is seldom a normative lingual expression. Most of these individuals also show a milder expression of other signs of RS[49,50].

- **Mildly affected females** — MECP2 mutations have been identified in females with a RS variant, mild learning disability, and even in a few women with no apparent symptoms who demonstrate extreme skewed X-chromosome inactivation[14,25].
- **Angelman-like RS** — These individuals show Angelman-like dysmorphism and behavioral characteristics, and have a refined hand function usually observed through manual stereotypes expressed in manipulating objects in an autistic manner. They usually show higher mobility traits, but less social involvement than most individuals with RS[51].
- **Males with RS** — Many males have been suspected of carrying the RS mutation even before the discovery of the RS gene[52,53,54], but none of them presented the full clinical characteristics of the disorder. Males meeting the clinical criteria for RS have been identified in association with a 47,XXY karyotype[55,56] and postzygotic MECP2 mutations resulting in somatic mosaicism[57,58]. Males with a 46,XY karyotype, however, can also be affected by mutations in MECP2. In later publications, several males have been positively identified as carriers of the RS mutated gene[26,44,59,60]. It seems that like females with RS, males with MECP2 mutations can present clinical manifestations in a variety of clinical phenotypes from neonates with severe encephalopathy[44] to adults with mental retardation[26,59].

## DIFFERENTIAL DIAGNOSIS

In 1997, Leonard et al. investigated the primary diagnosis of 82 girls who were later positively diagnosed with RS[6]. Despite the fact that more than 50% of individuals with RS shows clear characterizing signs of stage II RS before the age of 15 months[61] (and the research population in Leonard's inquiry has visited the physician at the relatively advanced age of 17 months), only 11 (13%) where diagnosed initially with RS. The rest of this sample (N = 71) were diagnosed with developmental delay or cognitive impairment (N = 42), hypotonic cerebral palsy (N = 14), autism (N = 9), and maternal anxiety (N = 6)[6]. To emphasize the distinction between those groups of patients, we present some characteristics that might assist in differential diagnosis.

**Rett Syndrome**

- Normal development up to 6–18 months.
- Progressive loss of skills with loss of speech and hand function.
- Ultimate level of functioning reflects profound mental retardation in all intellectual domains according to conventional intellectual testing.
- Acquired microcephaly, growth retardation, and decreased weight gain.
- Stereotyped hand clasping, hand wringing, or hand washing movements are present in every case; tooth grinding and breath holding are also common.
- Gait deteriorates and is characteristically straight kneed, wide-based ataxic; some may become nonambulatory.
- Language is mostly absent.
- Eye contact is present.
- Most children show little ability to perform hand function.
- Seizures develop in 75–80% of the population between ages 1 and 3 years.
- Behavior modification techniques do not significantly impact target behaviors.
- The syndrome occurs mostly in females.

**Early Infantile Autism**
- Onset before 30 months of age, and often from early infancy.
- Loss of previously acquired skills does not occur; generally slower rate of development.
- Level of cognitive functioning is varied; 40% have IQs < 50 and 30% have IQs > 70; additionally, there is scatter among intellectual domains, with more efficient performance in manipulative or visual spatial skills and immediate memory, while verbal abilities and abstract thinking are found to be more impaired.
- Physical development is usually normal.
- Stereotyped behavior is more varied, mainly displaying behaviors including some form of self-stimulation and object manipulation.
- Gross motor skills are mostly and sometimes impressively superior.
- Language may be absent; if speech is present, peculiar speech patterns, such as immediate and delayed echolalia and pronominal reversal, are observed.
- Eye contact with others may be avoided.
- Ritualistic behavior may involve odd objects manipulation in a stereotyped way; other obsessive behaviors might exist.
- Seizures develop in 25% of the population in adolescence and early adulthood.
- Children respond to behavior-modification techniques.
- The disorder occurs in males and females, with a 3:1 ratio.

**Cerebral Palsy**
- Onset from birth, unless the result of a specific postnatal insult up to the age of about 1 year.
- Delayed acquisition of normal developmental milestones and skills; physical handicap interferes with motor development.
- Level of functioning is varied; range of IQ is from normal to below normal and will vary with compounding factors.
- Physical development may be influenced by the extent of motor involvement and additional medical complications.
- Stereotyped behavior is not typically a characteristic of this diagnosis.
- Gross motor development is delayed or abnormal; ambulatory status is somewhat related to the type and severity of cerebral palsy.
- Receptive language may be much better than expressive; expressive language ability is related to the extent of motor involvement.
- Eye contact is present.
- Ability to manipulate objects may be influenced by the extent of motor involvement.
- Seizures are present in 86% of spastic quadriparetic cerebral palsy, 12% of athetoid cerebral palsy.
- Behavior modification may be appropriate for specific situations.
- The disorder occurs slightly more frequently in boys than girls.

## NEUROPATHOLOGY IN RETT SYNDROME

When considering the vast clinical/neurological signs displayed by individuals with RS, the pathological findings within the neural system of individuals with RS are but few[62]. The brains of individuals with RS are small and closely packed with neurons. The fact that the brain weight of individuals with RS is 60–80% of expected weight in age-matched females without RS[63] may explain the microcephaly diagnosed in this population. Decreased dendritic spines and arbors have been noted[64]. The closely packed cells at the cortex, thalamus, basal ganglia, amigdala, and hippocampal areas resemble the brain of a young infant[65]. In individuals with RS entering their second decade of life, mild loss of neurons and moderate nonspecific gliosis is observed. Most of the basal ganglia structures seem relatively normal, yet the severe movement disturbances observed in RS are attributed to the Substantia Nigra abnormality[63,66]. At present, it is postulated that RS pathology is derived from a problem in "synapse migration". This means that the formation of neural connections throughout the cerebrum is deficient, explaining both the low brain activity and the low postsynaptic innervations observed in different receptors[67,68]. Lately, MECP2 was found to influence the expression of BDNF (brain-derived neurotropic factor), which is related to synaptic organization, brain plasticity, and long-term memory[69,70].

## CLINICAL CHARACTERISTICS OF THE INDIVIDUAL WITH RETT SYNDROME

Individuals with RS display a wide variety of clinical manifestations; most of them are listed below.

- **Ambulation** (crawling and walking) — Most children with RS begin to crawl later, and usually display abnormal forms of crawling. Most of them will not show customary reciprocal crawling; some of them compensate by scooting, bunny hopping, or rolling[71]. In 85–50% of children with RS[12,72 accordingly] walking is achieved. Individuals who do achieve walking ability usually gain the ability later than 19 months of age, with an anecdotal report from parents regarding initiation of walking of their daughter at age 16 years. The regular walking pattern of individuals with RS is a wide-based, straight-kneed ataxic gate. Many of them start toe walking at later stages, which necessitates intense physical therapy or orthopedic intervention. At the second or third decade of life, 85–33% of the individuals that have achieved walking might lose the ability to ambulate[12,73 accordingly].
- **Apraxia** — Apraxia is a broad term relating to situations in which one is having difficulties in performing a motor act on demand or in learning a new function. It is derived from a deficit in sensory (tactile, proprioceptive, vestibular) information processing, causing a skewed body scheme that disables the child's motor planning[74]. This difficulty affects the individual in all functional areas (feeding, ambulating, speech, hand function). The presence of apraxia and its immense functional effect suggests that the individual with RS should be treated in accordance to guidelines of intervention with individuals with apraxia[2,75,76].
- **Balance and coordination** — When ambulating, the individual with RS has to confront her unreliable sensory processes (apraxia), her fear of movement, her fluctuating muscle tone, and the poor muscular control attributed to ataxia. The accumulation of such challenges inflicts severe functional limitations on the individual with RS. Therefore, many of them will find it hard to handle tasks, such as ascending or descending staircases, walking on uneven terrains, running, and more. On the other hand, in some cases, children with RS have been known to use a trampoline, ride a bicycle, ski, or use skates. A daily, low-intensity, short-termed treadmill program has been found to improve functional ability of children with developmental disabilities[81] including four girls with RS[82]. These reports and the assumption that the fourth stage of RS might be prevented in some cases if intervention is appropriate and intense enough[83], advocate the relevance of early intervention and intense programs for this population.
- **Breathing abnormalities** — Most (84%) individuals with RS present some form of breathing abnormalities, such as apnea, hyperventilation, breath holding, and bloating, usually from stage III of the disease[77]. These incidents are apparent during waking hours alone[78]. The underlying assumption is that the immature regulatory system supervising the breathing mechanism can supply the individual with RS needs during sleep, while breathing irregularities represent the incapability of the system to cope with functional changes necessary during daytime activities[71]. It is important to note that breathing irregularities during sleep are a sign of a respiratory problem and these should be examined by a physician since they might indicate an acquired illness[71]. According to Julu and associates[79], individuals with RS show 13 types of breathing irregularities divided into 4 main categories. Some of those respiratory irregularities respond to medication, others do not. An expert in the field of RS should be consulted in regard to the child's breathing pattern and the proper care advised[80]:

1. Breath holding
2. Forced expiration
3. Feeble\shallow breathing — The patients with this type of breathing are showing low PO2 and raised PCO2 that can lead to repeated episodes of exaggerated simultaneous increases in cardiac sensitivity to baroreflex, cardiac vagal tone, and blood pressure. During such incidents, the functional indices of the whole brain stem were simultaneously and momentarily increased; a situation termed by the authors as "brain stem storms". Such sudden large increases in vagal tone with poor ventilation, raised carbon dioxide, and hypoxia carry a risk for cardiac arrest[79].
4. Valsalva breathing — Individuals with RS presenting this type of breathing are more susceptible than other individuals with RS to a phenomenon termed "brain stem shutdown" (characterized with a drop in arterial blood pressure). In severe cases, the brain stops controlling the heart rate and the pacing of the heart is transferred to the internal pacer. At this point, the person will look lifeless, therefore proper diagnosis and treatment is due.

- **Body scheme** — Due to sensory imbalance of individual with RS and immaturity of cerebral functions, it is probable that their body scheme is incomplete. This could be concluded from the apraxia shown by many of the persons with RS and from the skewed midline[84,85,86] presented by some. Their fear of movement is probably caused by this same problem. Experiencing such sensory uncertainty on a continuous basis could lead to a sense of fear and insecurity, leading to immobility. To reduce some of the anxiety accompanying the transfers of the individual with RS, the application of a daily program is recommended that will supply intense proprioceptive input (deep pressure) in order to help the individual with RS to build up her sense of orientation[2,86].
- **Bruxism** (teeth grinding) — Teeth grinding during waking hours appears in 53–95% of individuals with RS. It is usually more intense at younger ages and disappears in adults[87]. Bruxism is typical in periods of excitement or anxiety. These movements may cause damage to incisors and canines mainly, but molars could be damaged as well[88].
- **Cognitive abilities** — Individuals with RS display functional abilities corresponding with severe-profound levels of intellectual disability. Nevertheless, conventional intelligence tests are based on hand function and linguistic abilities of the participants (two of the weakest traits of individuals with RS), thereby putting into question the appropriateness of those instruments for this specific population. Regardless of findings by conventional intelligence evaluation tools, it is apparent that these individuals possess learning ability[89,90,91,92] and that those skills can be enhanced through proper motivational factors. The findings also show that learning in individuals with RS is maintained after a washout period[91]. It is also apparent that learning, including literacy skills[92], is possible in this population throughout adulthood[87].
- **Communication** —The primary development of the child with RS leads to the belief that their communicative attunement and intentionality can be kept and used with alternative communication[93]. Individuals with RS show the emotional and social need to communicate, yet this need is coated by their disabilities[94], making their communicative acts hard for their human environment to interpret. Nevertheless, when their motivation is high enough and the human surrounding attentive, the individual with RS will be able to convey her needs and wants, greatly improving her quality of life[95,96]. Some individuals with RS use pictures, body language, and gestures[87] as their communicative acts. The use of AAC (alternative and augmentative communication) can compensate for the severe communication difficulties shown by users[97] and is therefore highly recommended for individuals with RS. It is recommended that every individual with RS should be evaluated for her communication skills (preferably by an expert in the field of AAC and RS) and the most appropriate communication approach will be recommended to her according to the user's skills and character[90].

- **Digestive system** — Many (74%) individuals with RS present gastrointestinal problems[98]. Intestinal problems detected in individuals with RS are gastroesophageal reflux (GER), bowel dysmotility, constipation, and functional megacolon. GER is more common in nonambulant girls and will present with regurgitation of food, vomiting, dysphagia related to esophagitis, chronic cough, and recurrent aspirations. Evaluating the gastroesophageal junction's function by milk scans and PH-metria is suggested in the more severe cases. Treatment includes antireflux agents, antacids, smaller and thickened feedings, and proper positioning. When paraprofessional and pharmaceutical intervention fails, surgery in the form of funduplication and gastrostomy might be suggested. One of the most common problems of individuals with RS is constipation. Severe and recurring constipation might cruelly impede one's quality of life. In extreme cases, fecal impaction, volvulus, and intussusceptions occur[99]. Diet including a high fluid and fiber intake with occasional use of stool softeners can help to prevent acute intestinal crises, and prevent or minimize the occurrence of constipation[2]. When those measures are ineffective in controlling constipation, Miralax (polyethylene glycol) has been found highly effective and is tolerated better than milk of magnesia. Recent findings from a large cohort have recognized significant gallbladder dysfunction, especially in children with RS younger than 20 years of age. The findings include gallbladder dysfunction and gallstones. These findings present a significant problem in RS. The frequency seems to be far greater than that in the general population of children[51], and medical evaluation and specific consideration should be taken.
- **EEG findings** — Certain EEG findings common to the disorder are not unique to RS, yet they are relatively specific and include slowing of the occipital dominant background activity with very frequent spike or sharp wave discharges mainly in central regions accentuated during sleep early in the course of the syndrome (usually during the regression stage). There is also loss of nonrapid eye movement (NREM) sleep characteristics. Later in the course of the disorder (end of stage III–stage IV), there is mainly slowing of background with decreased epileptiform activity. Video/EEG monitoring reveals frequent episodes of apnea and hyperventilation, laughing, screaming, and vacant staring spells with no consistent EEG correlate[100].
- **Emotional reaction** — The child with RS grows up from birth experiencing common parental affection as any other child, in a way that supports the development of a normative emotional state[101]. At the beginning of stage II of RS, the child experiences extreme disorientation[87] and deterioration, leading to frustration, anger, sadness, fear and surprise, and a sense of loss. This loss of control over her body and mind is unapprehendable and can cause the child to burst into angry rage or to give in to an emotional withdrawal and apathy. At the end of that turbulent stage, the end of functional deterioration, the child can regain some of her emotional balance that can be observed by calmer disposition. Nevertheless, the person with RS will be dependent on others in many of her daily routines and the compliance, understanding, and faith of her human milieu in her abilities will affect her behavior and her emotional expression for better or for worse[102]. Negative changes in emotional expression (crying, moody disposition) have been attributed to constipation, hyperventilation, bowel pain, a noisy environment, an excess of stimuli, and temperature changes. These changes can shift from laughter to dissatisfaction and back again within seconds, or they can last for a few minutes or up to several hours. The bottom line is that the individual with RS has a full emotional variability[101] and that her emotional state originates from her daily encounters and internal body signs. Her difficulties in sending out clear communicative messages make it the responsibility of the caretaker to get to know the child, to decipher the origin of her unpleasant disposition, and to try and resolve her situation, thereby answering to her emotional well-being as well.

- **Epilepsy** — Epileptic seizures have been reported in 30–90% of individuals with RS[11,12,100]. Seizures occur more frequently when the disorder stabilizes (at the end of stage II, usually between 2–5 years of age), with a higher tendency to seize during a febrile illness. Generalized tonic-clonic seizures and partial complex seizures are the most common[103]. Additional manifestations of seizure activity include simple partial seizures and absences (including atypical absences and myoclonic events). Focal electrographic seizures are usually associated with focal clonic activity, head or eye deviation, and sometimes apnea[104]. Episodes imitating epileptic events might actually be related with motor activity, such as twitching, jerking, and trembling, or a cardiac arrhythmia associated with a prolonged QT interval[104]. These events are not correlated with epileptiform activity on EEG. On the other hand, clinical events accompanying EEG epileptiform activities (such as absence episodes or flexor spasms) are not always recognized as seizures by the parents[104]. EEG video-telemetry reveals the fact that "observed attacks are in actuality events of breath holding and should not be medically controlled"[100]. Use of video EEG for further defining these episodes or at least discussing them with an expert in the field of RS will prevent disuse of antiepileptic drugs. Evidence shows that epileptic activity in RS is reduced and is mostly easily controlled in individuals reaching adolescence[104,105]. In many cases, seizures are easily controlled by medications in individuals with RS, yet in some they might be intractable. Common pharmacological interventions in case of epilepsy in this population include Valproic acid, lamotrigine, topiramate, carbamazepine, and recently Keppra (second author's personal experience). Phenobarbitone and to a lesser extent benzodiazepines display a severe effect on the level of alertness and responsiveness of the client with RS to a point of sudden pseudomotor deterioration. Their use should be avoided as much as possible.
- **Feeding** — Individuals with RS usually enjoy eating. They present a developed sense of taste with obvious food preference[87]. It was established that frequent small meals during the day with added carbohydrate foods can support growth as well as weight gain in individuals with RS[71]. Due to their functional limitations, meal time is usually prolonged. Individual with RS are very slim, averaging 17.5 BMI (the normal BMI for their peer group is 21–26)[98], apparently due to altered energy balance[106]. Furthermore, it has been found that many individuals with RS show mild to moderate malnutrition[71], and due to their feeding difficulties, their caloric intake is 70% of the norm[107]. Feeding problems are common in RS[99,108]. Some difficulties that might impair the meal-time experience of the individual with RS are spitting, immature chewing patterns, exaggerated gag reflex, reflux, swallowing difficulties, and intestinal problems[109]. In order to maximize the enjoyment of the individual with RS from experiences associated with eating, the physical and human environments of the person with RS should be individually evaluated and adapted[2]. In cases where nutrition supplements do not supply the person with RS full caloric intake, a gastrointestinal tube might be considered. After such a surgical procedure, individuals with RS have been found to gain weight, resume growing rate, present less agitation and irritability, and function on higher activity levels[110,111].
- **Growth failure** — Most individuals with RS show a decrease in growth and weight gain presented in average BMI measures at 17.5[98]. Growth failure is detected in 85–90% of individuals with RS and the situation worsens with age[11,71,110,112]. This situation, if not taken care of, might even develop into a lethal malnutrition. The individual with RS healthy appetite and long meal duration, which might create the illusion that the individual is consuming a high caloric intake. It is postulated that this growth arrest is derived from poor coordination of both the oropharyngeal and gastroesophageal system, which results in poor caloric intake and high energy expenditure[106,107,108,109]. In relation to this topic, it is emphasized that persons with RS should be supplied with a fat- and carbohydrate-enriched diet, and with vitamin and mineral supplements. A continuous supervision by a dietitian acknowledged in RS is highly recommended. In some cases where nutrient uptake is low and conventional food supplements are not enough to enhance the child's development, a gastrointestinal tube is recommended. As far as we know, up until today, all parents who agreed for this operation to be performed were very pleased with the results.

- **Head circumference** — At birth, head circumference is usually found on the lower normal percentile[24]. Between 2–4 or 5–12 months postdelivery[29 and 28 correspondingly], head growth gradually decreases, resulting in true or relative microcephaly[113]. Stenbom and associates[114] reported a correlation between head circumference and functional abilities of children with RS at 1 year of age, but those findings were preliminary and were not supported by other research groups.
- **Hearing** — Due to communication limitations of individuals with RS, it is a complex task to perform a conventional hearing test. Nevertheless, it has been widely observed that the sense of sound is extremely developed in individuals with RS. It is common knowledge that they love music[115,116,117,118] and they have been found to remember special sounds and have the ability to connect them to specific events. They love the sound of children singing and the whisper of adults[87], and music was established as a key motivator for learning for this population[89,90]. In a single report, a slight to moderate loss of hearing was found in 17.3% of the participants. Hearing loss was mainly found in adult participants with RS and was attributed to the use of anticonvulsive medication[119].
- **Hyperkinetic movement** — Hyperkinetic movement is characteristic of the individual with RS who possesses the ability to ambulate. It is believed that the individual with RS knows her goal and direction of movement, but that her movement interfered by ataxia and apraxia is perceived by the external observer as senseless/aimless roaming[71].
- **Lack of hand use** — Most children with RS acquire some form of hand function in their postgestational period. Such hand function could be grip, self-feeding, and some parents even report the ability of their children to use the finger dial on the phone or to flip pages of a book at a very young age. From about 8 months and up to 4 years, these children gradually lose hand function. It was found that 33.6% of children with RS have a left hand preference, 40.7% show right handedness, and 25.7% show no preference in hand use[125]. Children with left handedness usually show a dominance transference to the right at age 7–8[87]. The assumption is that poor functional abilities of individuals with RS are not derived from lack of ability, rather from the combination of apraxia and lack of experience[126]. Hence, despite the deterioration of functional hand use, individuals with RS can and should be taught repeatedly to acquire new hand skills[127].
- **Language** — The development of language in infants with RS is extremely rare. At infancy, vocality as well as protospeech are slower to develop and are usually poorer than would be expected of babies without RS. In our experience, some girls retain the ability to express a few words; this ability is usually expressed in stressing (“afraid”, “mammy”) or highly excitatory situations (“cookie”, “dog”). We believe that most individuals with RS understand the meaning of many words, enjoy listening to children and adults talk, and are extremely fond of stories. When given the chance, many individuals with RS can acquire literacy to the level of a few tens of words. The preserved speech variant (PSV) is found in 1–4% of all individuals with RS[49,128]. These individuals have the ability to speak, usually in an odd, partial vocabulary.
- **Life expectancy** — Due to the rarity of RS and its relative novelty to the scientific world[4], very little is known about long-term prognosis and life expectancy. While there are a few women in their 40s and 50s that have RS, there have been too few women studied to make reliable estimates beyond age 40. The average life expectancy of a person given the diagnosis of RS may exceed 47 years[129]. Females with RS typically survive into adulthood, but the incidence of sudden, unexplained death is significantly higher than in controls of similar age[130]. This sudden death may be due, in part, to the higher incidence of longer QT intervals, T-wave abnormalities, and reduced heart rate variability in RS[131,132].but can also be related to SUDEP (sudden death due to epilepsy)[133] or other mortality causes due to "complications of Rett syndrome"[134], such as breathing abnormalities[79].

- **Muscle shortening —** Muscle shortening is common in individuals with RS presenting high muscle tone. It should be noticed, however, that low muscle tone initially shown by individuals with RS might gradually be increased with age[73]. The first muscle influenced by the high tone is usually the gastrocnemius (attached to the heel cord). In that case, the individual will start to toe walk. In severe cases, the person with RS can lose her ambulation abilities. This phenomenon can be addressed by the physical therapist through a daily standing program, by using specially designed active splints, or through serial casting[2]. In severe cases, the orthopedic surgeon can suggest botulinum toxin treatment or surgery. Other muscles that tend to shorten are the hip adductors and, in wheelchair-bound individuals, the hip flexors. Another group of muscles in danger of shortening are the anterior muscles of the shoulder girdle. This happens due to rarity of hand function in this population and the constant hand mannerisms usually performed at the midline at chest level[135]. On routine examination, the physical therapist and the orthopedic surgeon should evaluate the above-mentioned muscles and, if necessary, implement an appropriate intervention program.
- **Muscle tone** — Individuals with RS present abnormal muscle tone. Most children with RS show a gradual change from very low muscle tone or even severe hypotonia at birth and during the first stage of RS. Gradually some of them (about 40%) will develop high muscle tone including spasticity and/or rigidity that will initiate from the shin muscles. About 30% will remain hypotonic and the rest will show fluctuating/distonic muscle tone[73]. Individuals with RS who show the least muscle tone (hypotonia) as infants, become the most rigid or spastic (hypertonic) in adulthood[72]. Such unbalanced muscle tone accompanying ataxia and apraxia will severely affect the person's posture and stability, thus severely harming her ability to function or self-ambulate. These difficulties necessitate intensive physical therapy intervention aimed at tone reduction and enhancement of functional capacities.
- **Orthopedic problems** — Over the years with the progression of RS, some of the following skeletal problems might accrue.
  1. Scoliosis — Severe cases of scoliosis appear in 83% of children with RS, while a deviation of the spine of at least 10 degrees can be detected in all other persons with RS[12]. According to Kerr[73], 90% will develop scoliosis and in 60%, it could be defined as severe. Scoliosis in RS can begin at 3–18 years of age. In some of the clients, worsening is evident between 5–15 years of age. When the spinal curve exceeds 50 degrees, surgery is advised by the orthopedic surgeon to alleviate difficulties in ambulation, spinal mobility, and respiratory function. An early diagnosis followed by an intense physical regime was found useful in regressing the spinal deformity in a child with RS[136], and prevention of the need to perform surgery in several others[109]. Intense physical therapy is recommended in this situation by all experts in the field of RS[124,136,137,138,139]. Corsets have not been found to be an efficient means to prevent or improve the progression of scoliosis[140], but they provide support and enable function since they help the individual to maintain erect posture. When the condition of the individual during the pre-surgery period is severe, most parents are in agreement with the results of the operation[141,142].
  2. Kyphosis — Kyphosis is more frequent in mobile individuals with RS. In most cases, it is trivial enough to evade intervention. However, due to the forward posture of the shoulder girdle of the individual with RS who is constantly occupied with stereotypical hand movements, it is advised to check the individual with RS condition during routine visitation to the orthopedic surgeon and to the physical therapist, and in case of muscle shortening at the anterior aspect of the chest, to recommend proper intervention[2].
  3. Hip instability — This orthopedic problem is not extremely common in individuals with RS; it tends to present itself in nonambulatory individuals[142]. Hip dislocation might be avoided through the use of preventive measures, such as a daily standing program, manually keeping the joint with full range of motion, serial casting, the use of hydrotherapy to strengthen the muscle around the joint, or by using botolinum toxin in specific injection sites[2].

4. Feet deformities — Represented by a deviation from the normal alignment of the feet, which is caused by asymmetry in muscle activation. It is common among wheelchair users and severely affected individuals with RS[142].

- **Osteoporosis/Osteopnia** — Osteoporosis occurs frequently in females with RS and decreased bone mineral density has been reported in very young girls as well as adults in comparison to controls[120]. It is assumed that this phenomenon results from poor bone formation. Due to osteoporosis, pathological fractures might occur[121]. As might be expected, fractures are more common in nonambulatory individuals, providing yet another reason to preserve the client's walking ability[122]. Antiepileptic drugs are also known as a contributing factor to osteoporosis due to their negative effect on Vitamin D absorption[123]. These findings support the need for routine checking of the bone density of individuals with RS from childhood, and to commence physical (such as intensive standing and walking programs[124]), nutritional, and medical intervention as preventive intervention when the situation necessitates such courses of action.
- **Pain sensation** — A decreased pain sensation in the population of RS was reported numerous times in the past[12,33,71,87]. Other neurochemical findings in this population support the possibility of a decreased reaction to pain. Kalmanchey[143] found reduced conduction velocity in peripheral neural pathways in individuals with RS. Witt-Engerstrom and Hagberg[144] found a reduction in number of dorsal root ganglia that serve as ascending pathways to painful stimuli towards brain pain centers. Budden et al.[145] reported high values of beta-endorphins in the spinal fluids of individuals with RS, moreover, low levels of P-substance (a neurotransmitter involved in relaying pain sensation), were also found in the neural systems of individuals with RS[146]. The accumulating evidence supports the notion that pain sensation is reduced in individuals with RS. Due to these findings and since verbal communication of individuals with RS is lacking, manual treatment should be performed with caution and each person with RS should be carefully checked for possible tissue damage with any change in mood or behavior.
- **Pregnancy and perinatal periods** — No common complications are known at those periods. A study by Huppke et al.[24] found that infants with RS are delivered with a normative, but rather small, head circumference and a normative, yet low, birth weight. This research also found that in 30 out of the 70 (42%) cases investigated, slight pregnancy complication could be reported; such incidence is not specific to RS.
- **Prolonged reaction time** — It is common knowledge that individuals with RS show prolonged reaction time[87,92,94,147,148,149,150]. Researchers in the field report that the response of the child occurred 30 sec and even longer after a request was presented to the participants[92]. The fact that the individual with RS might be intensely occupied with breathing irregularities and vaguely gaze at the object to be manipulated might be wrongly interpreted as if she misunderstood the request or lost interest. All these behaviors are the person with RS way of preparing for appropriate action. Individuals with RS should be allowed the amount of time they need to succeed in accomplishing a task. With proper motivational factors, reaction time could drop considerably[91].
- **Sensory system** — The sensory system of individuals with RS was never thoroughly investigated; nevertheless, accumulating reports suggest that this system is not functioning in a normative manner in this population. Lindberg held an educated observation[87] and, with the aid of parental reports, suggested that individuals with RS show difficulties in receiving, deciphering, and processing of external stimulus. In the extremely turbulent second stage of RS, many individuals show extreme agitation regarding external stimuli through diverse sensory modalities. At that stage, a quiet, reduced-stimulation, relaxing atmosphere should be preferred[151]. The tactile system has been reported as oversensitive in individuals with RS especially around the mouth and face, which can be observed in sensitivity to teeth brushing and resistance to different food textures. In our experience, some children react negatively (i.e., crying, face frowning, hand pulling) to proprioceptive input over the forearms. Taste and olfactory senses, on the other hand, do not seem to be affected[71] despite sporadic reports[152]. The auditory and visual systems are considered the paramount sensory input for individuals with RS[36,71]. Many individuals with RS use the visual system as their main communication path, pointing at desired directions and communication symbols. This system was found to be preserved and functions at a reasonable level even among the highly affected individuals up to late adulthood[12], except for a few cases showing myopia (shortsightedness) and hyperopia (farsightedness)[71]. The auditory system is a very active pathway that enables the individual with RS to enjoy the sounds of music and human voices so stimulating for them. Somatosensory and auditory stimulations were tested several times in individuals with RS and were found within the norm or slightly deviated from different control groups[153,154,155].

- **Sleep patterns** — Individuals with RS show significant sleep problems, especially during stages II–III of the disorder. Some individuals find it difficult to fall asleep; some might do so only at extreme exhaustion and might need the use of medication[157,158,159]. In some instances, children can wake up with idiopathic crying or laughing spells. They might wake up in the middle of the night to be fully alert and active, and as a result become sleepy during the next day. These spells usually occur in clusters of several days to weeks and subside spontaneously[160,161]. In addition, nocturnal seizures, GER, and constipation are possible causes for disturbed sleep, and should be diagnosed and treated accordingly. Children with RS sleep more than their peers and this tendency intensifies with age[160,161]. In contrast to individuals without RS, individuals with RS have been found by some researchers to continuously go over repeated short cycles of deep and REM sleep. It is hypothesized that this pattern is generated due to dopaminrgic\serotonergic system abnormality[158,159]. Various solutions have been found to help the individual with RS and her family to achieve continuous night sleep: medication[157] including melatonin, barbiturates, benzodiazepines, and other sedatives. Other more traditional methods might include: cooling or warming of the room (according to the preference of each individual), deep massage, a white noise machine, a water bed, a car ride, soft music, hot bath before sleep time, maintaining high activity levels throughout the day, and more[71, p. 123].
- **Stereotypical hand movements** — Manual mannerisms are apparent in all individuals diagnosed with RS. They appear simultaneously with loss of hand function and are typically performed at body midline, at chest level. These vigorous movements are apparent in 30–72% of the individual's waking hours[37]. These movements intensify when the person with RS is exited\hungry\tired and they stop only when she is asleep. The movements are not symmetrical[162] and can be performed as hand clasping, hand wringing, or hand washing movements, sometimes touching the mouth and tongue. Usually, the individual who shows more intense hand stereotypical movements also shows better manual functional abilities. These movements are involuntary, do not include the use of objects (beside Angelman-type RS), and are not used for protection or as a response to a sensorial need[37]. Voluntarily stopping the hand movement is usually very difficult for the individual with RS, although some can withdraw from those continuous movements for a short period on demand. It is speculated that the stereotypical movements are caused due to neurochemical imbalance in the dopaminergic, monoaminergic, and cholinergic systems[163,164]. Despite the fact that in late stages of RS the stereotypical movements subside, they can still be observed[87].
- **Vision** — Despite the fact that the eyes are a major communication organ in individuals with RS, 50–100% of them display slight visual problems[87,156 accordingly], as well as unorthodox ways of using the eyes. They tend to look at an object sideways before focusing on it. This phenomenon was defined by Lindberg[87] as "Eyes who can see without looking" (p. 25). About 80% of individuals with RS show a slight strabismus (cross-eyed) at a young age (during the first decade of life), which disappears at older ages[2].

## MANAGEMENT

There is no known medical intervention that will change the outcome of patients with RS. Hence, the medical intervention is symptomatic. Intervention in RS should provide support and comfort to the individual with RS and her family. A multidisciplinary team approach is advocated[11,165,166], aimed at maximizing each person's abilities and facilitating any skills that may be emerging. Psychosocial support for the families, development of an appropriate rehabilitation/education plan, and assessment of available community resources should be provided. Parent support groups are an elementary aspect in providing support for families of individuals with RS[166].

### Pharmaceutical Intervention

From the medical viewpoint, pharmacological treatments for RS have included several drugs that were suggested and investigated in regards to alleviation of symptoms of this disorder:

- L-carnitine was tested in the past in a double-blind trial as a possible therapeutic agent. Despite caregiver reports on improvements in patients' general well-being, significant functional improvements could not be observed by the researchers[167].
- Naltrexone, an oral opiate antagonist, was investigated due to the fact that elevated opioids had been observed in the CSF of persons with RS. Although it decreased breathing dysrhythmias and had some sedating properties, its efficacy is controversial due to reports on deterioration of the course of the RS disorder[168].
- Risperidone or selective serotonin uptake inhibitors (other possible medications are chloral hydrate, hydroxyzine, or diphenhydramine along with melatonin) have been somewhat successful in treating agitation in a group of children with PDD[169].
- Carbidopa/levodopa has been tried for reducing rigidity in other populations than RS[170].
- Magnesium was introduced for individuals with RS to reduce the episodes of hyperventilation[171].
- Melatonin was found to possess some ameliorative effect on sleep disturbances[157].
- A trial involving dietary supplementation with folate and betaine that was based on the assumption that some alleles of the affected genes could be altered by dietary methyl supplementation did not show any significant benefits for individuals with RS[51].
- Folinic acid was suggested and found to normalize 5-MHTF and 5-HIAA levels in the CSF in four patients with RS by Raemakers and associates, thereby leading to partial clinical improvements in those patients. Therefore, the researchers suggested that folinic acid should be used to increase folate metabolites in the CSF of persons with RS[172]. Those finding were not confirmed by Neul and associates[173].
- An ongoing study conducted by Naidu and associates is testing the efficiency of dextromethorphane as a glutaminergic blocker. Preliminary results show some benefits in epilepsy control and general well-being in part of the participants.
- In accordance with recent reports demonstrating that BDNF overexpression in the brain tissue of individuals with RS reduced disease progression, Tsai[174] has suggested a favorable manipulation of BDNF expression in the brain through the use of lithium and other antidepressants, since they have been found to increase central BDNF levels or signaling in human as well as animal studies[174]. No clinical trial was performed yet to assert this hypothesis.

## Medication that Should be Avoided in Persons with Rett Syndrome

Prolonged heart rate–corrected QT values have been reported in association with RS[131,175]. In order to prevent electrocardiogram QT abnormalities and cardiac arrhythmias, the use of some drugs should be avoided by individuals with RS. Theses are prokinetic agents (i.e., cisapride), antipsychotics (i.e., thioridazine), tricyclic antidepressants (i.e., imipramine), antiarrhythmics (i.e., quinidine, sotolol, amiodarione), anesthetic agents (i.e., thiopental, succinylcholine), and antibiotics (i.e., erythromycin, ketoconazole)[65,176].

## Allied Health Professional Intervention

Occupational and physical therapy are crucial for maintaining function and preventing deterioration, while improving the well-being of the individual with RS and her family. Augmentative communication is essential to enhance the child's control over her environment. Music and music therapy are key elements in every intervention applied for individuals with RS; therapeutic horseback riding, swimming, are also beneficial[99]. The above-mentioned interventions would be an advantage to the individual with RS if a therapeutic dialog is kept between different professionals, and if a cooperative multidisciplinary milieu is present[166].

## Physical Therapy

The clinical characteristics of RS include the expression of several challenging physical disabilities. Scoliosis is found in approximately 85–65% of girls with RS[12,109]. Physical therapy intervention has been found to reduce the progression of scoliosis[136]. Some girls require bracing, while others require surgical intervention[109,138]. Increased tone in the Achilles tendon is one of the earliest manifestations of onset of rigidity, usually followed by toe walking. It is important to maintain ambulation and conventional physical therapy intervention methods can keep the Achilles tendons stretched[2,84,109,124]. Bilateral ankle foot orthoses might be considered to prevent foot deformities, maintain foot alignment, and keep the heel cords lengthened. Botolinum toxin injections are a possibility[141]. Versatile and comprehensive physical therapy interventions can help the individual with RS and her family reduce and overcome many of the debilitating features of this disorder[2].

## Occupational Therapy

Common RS difficulties must be addressed by a knowledgeable occupational therapist. Such problems could include the sensory abnormalities presented by individuals with RS, low initiative drive, hand stereotypical movements, the complex manual function, and short attention span. Regulating sensory imbalance of these individuals could be performed using the Snoezelen approach[151,177] or the sensory integration approach[178]. Low initiative drive might be due to the enormous challenges every action opposes on the apraxic, distonic, and ataxic individual with RS. Enabling the environment by using switches to operate daily apparatuses and continuous training in challenging situations might help the child to overcome some of her barriers[2]. Overcoming the repetitive hand mannerisms and achieving function can be accomplished by different approaches. Some therapists favor hand restraints that inhibit the repetitive and purposeless hand movements. Reduction of those hand mannerisms should be performed, if possible, by the use of soft splints and they are occasionally helpful in training specific hand skills, such as self-feeding and communication. These methods have been found helpful to decrease agitation and self-injurious behaviors in some individuals with RS[109]. Due to individual versatility of the manifestation of RS, the effect of splinting should be individually evaluated for each client and the administration of the device handled accordingly. Attention span could be increased by the use of quiet environments when individual intervention is performed. Involvement in various activities such as eating, operating musical instruments, and the use of computer programs are all recommended and could be pursued by the therapist.

## Speech Therapy

Research and information about the cognitive and communicative potential of females with RS is scarce, and sometimes show contradicting and indefinite data[94,149,150,179].

It has been indicated that people with RS communicate by using nonsymbolic means, such as gestures, vocalizations, and body positioning[33]; by establishing positive contacts with people's faces/eyes; and reacting with smiles as a positive answer[180].

The loss of expressive language in the majority of individuals with RS necessitates the intervention of a speech pathologist experienced with AAC devices. Such aids are used for making choices to empower the person with RS and facilitate environmental control. Alternative forms of communication are communication boards, technical devices, commonly used gestures, eye pointing, body language, and hand pointing. Each individual with RS’s abilities needs to be recognized by their close human environment and encouraged[91].

## Music Therapy

Females with RS have been observed as very responsive to music[116]. When music was used therapeutically by trained practitioners, this intervention was shown to promote and motivate their desire to interact and communicate with their surroundings, as well as to develop their cognitive, affective, sensorimotor, and physical skills[73,87,116,117,118,181,182,183,184,185,186,187].

Current knowledge of the use of music therapy with the person with RS suggests that music therapy can stimulate many aspects of development including choice making, enhanced vocalization, function and hand usage, improved eye contact, and open channels for emotional and communicative expression[117,118,137,181,182,183,184,185,188] and learning[91].

## Hydrotherapy

Individuals with RS face difficulties that affect them in many daily situations. In order to ease them into the physical treatment, they shall probably need to receive some kind of mediation throughout their lives. One of the favored milieus for persons with RS is water. Warm water helps reduce spasticity and softens rigid tissues. In addition to supporting the client’s unbalanced posture, water provides the individual with RS with massive stimulus. It assists in calming one who is afraid of motion; in the water, the individual with RS is able to move more slowly, easily, and freely, without the fear of falling and hurting herself[189,190].

Possible intervention goals for the individual with RS through aquatic therapy correspond with general therapeutic goals, such as enhancing relaxation[151,189,190]; improving hand functions; maintaining and improving transitional skills[85,86,191]; regulating breathing patterns; preventing potential orthopedic problems and treating existing ones[139,189,190]; reassembling “midline”[84]; improving equilibrium coordination, and communication; regulating the sensory system; improving social skills; and supporting and augmentating physical activity[189,190].

## Hippotherapy

Hippotherapy is part of a range of experiences known as "equine-assisted activities". Hippotherapy refers to the use of the movement of the horse by a skilled and licensed professional to address impairments, functional limitation, and disabilities in persons with nerve, muscular, skeletal, vestibular, and/or behavioral problems, as part of an integrated treatment approach geared to improving function.

Equine-assisted movement experience provides the participant with a variety of sensorimotor experiences through changes of position on the horse. A variety of goals that can contribute to most individuals with RS and addressed by hippotherapy might include among others: produce active mobilization of the pelvis, lumbar spine, and hip joints; develop body symmetry and awareness; equilibrium reactions improve posture, balance, coordination, motor-planning, strength, and/or endurance, timing, and rhythm.

## SUMMARY

In this review, clinical characteristics of individuals with RS were introduced. The intent was to update the familiarized reader with the latest knowledge and to introduce this syndrome to individuals taking their first clinical steps in "Rettland". We hope that this article will add to the reader's existing knowledge, thus advancing the quality of knowledgeable management received today by individuals with RS around the world.
